# The impact of critical illness on the expiratory muscles and the diaphragm assessed by ultrasound in mechanical ventilated children

**DOI:** 10.1186/s13613-020-00731-2

**Published:** 2020-08-27

**Authors:** Marloes M. IJland, Joris Lemson, Johannes G. van der Hoeven, Leo M. A. Heunks

**Affiliations:** 1 Department of Intensive Care Medicine, Radboud University Medical Center, Radboud Institute for Health Sciences, Nijmegen, The Netherlands; 2 Department of Intensive Care Medicine, Amsterdam UMC, Location VUmc, Postbox 7057, 1007MB Amsterdam, The Netherlands

**Keywords:** Expiratory muscles, Diaphragm, Mechanical ventilation, Children, Ultrasound

## Abstract

**Background:**

Critical illness has detrimental effects on the diaphragm, but the impact of critical illness on other major muscles of the respiratory pump has been largely neglected. This study aimed to determine the impact of critical illness on the most important muscles of the respiratory muscle pump, especially on the expiratory muscles in children during mechanical ventilation. In addition, the correlation between changes in thickness of the expiratory muscles and the diaphragm was assessed.

**Methods:**

This longitudinal observational cohort study performed at a tertiary pediatric intensive care unit included 34 mechanical ventilated children (> 1 month– < 18 years). Thickness of the diaphragm and expiratory muscles (obliquus interna, obliquus externa, transversus abdominis and rectus abdominis) was assessed daily using ultrasound. Contractile activity was estimated from muscle thickening fraction during the respiratory cycle.

**Results:**

Over the first 4 days, both diaphragm and expiratory muscles thickness decreased (> 10%) in 44% of the children. Diaphragm and expiratory muscle thickness increased (> 10%) in 26% and 20% of the children, respectively. No correlation was found between contractile activity of the muscles and the development of atrophy. Furthermore, no correlation was found between changes in thickness of the diaphragm and the expiratory muscles (*P *= 0.537). Decrease in expiratory muscle thickness was significantly higher in patients failing extubation compared to successful extubation (− 34% vs − 4%, *P *= 0.014).

**Conclusions:**

Changes in diaphragm and expiratory muscles thickness develop rapidly after the initiation of mechanical ventilation. Changes in thickness of the diaphragm and expiratory muscles were not significantly correlated. These data provide a unique insight in the effects of critical illness on the respiratory muscle pump in children.

## Background

The expiratory muscles are an essential component of the respiratory muscle pump. When the load imposed on the diaphragm increases, accessory inspiratory muscles and expiratory muscles are recruited to meet metabolic demands. Numerous studies have demonstrated that critical illness has detrimental effects on the diaphragm, both in adults and children [1–5]. In children, diaphragm atrophy developed within a few days after initiation of mechanical ventilation and affected almost 50% of these children [4, 5]. Low diaphragm effort (i.e., ventilator over-assist) was associated with a higher rate of extubation failure in these children [3]. However, the effects of critical illness on the expiratory muscles, both in critically ill adults and children have been largely neglected in the literature so far. This is surprising given the important role of the expiratory muscles not only in breathing [6–9], but also in airway clearance.

Therefore, the aim of the current study was to determine the impact of critical illness on the most prominent muscles of the respiratory muscle pump, especially the expiratory muscles and the diaphragm. In addition, we aimed to investigate the correlation between changes in thickness of the expiratory muscles and the diaphragm.

## Methods

### Study design and population

This longitudinal observational cohort study was conducted at a pediatric intensive care unit (PICU) of a tertiary hospital between November 2016 and February 2018. The institutional ethical committee approved the study protocol and informed consent was waived due to the non-invasive nature of the study and negligible risks. The study was performed in accordance with the ethical standards of the Declaration of Helsinki.

A convenience sample of 35 patients was selected, in line with similar previous observational studies in the pediatric population [3, 4, 10]. Patients were eligible for inclusion if aged between 1 month and 18 years, initiation of mechanical ventilation < 24 h of admission and an expected duration of invasive mechanical ventilation for ≥ 24 h after inclusion. Exclusion criteria include past medical history of neuromuscular diseases, congenital diaphragmatic hernia, known diaphragm paresis, chronic respiratory failure and receiving mechanical ventilation > 48 h in the 6 months before enrollment. As the ultrasound measurements were conducted by a single scientist (MIJ), inclusion was dependent on the scientist and availability of the ultrasound device. For this reason not all eligible patients could be included. To rule out any inclusion bias, we therefore performed a secondary analysis.

Patients were ventilated (Servo-i; Maquet, Sweden) according to the principles of protective lung ventilation according to the recommendations from the Paediatric Mechanical Ventilation Consensus Conference [11]. This means that the settings of the ventilator were aimed as follows: (1) tidal volume was set between 5 and 7 ml/kg; (2) plateau pressure was limited ≤ 30 cmH_2_O; (3) positive end-expiratory pressure (PEEP) was initially set at ± 5 cmH_2_O and if needed titrated for improvement of oxygenation; (4) the inspiratory time and respiratory rate was set depending on the respiratory system mechanics with avoidance of flow end-inspiratory or expiratory flow interruption; (5) with restored respiratory drive, patients were switched to pressure support ventilation with flow triggering (default setting). All patients were intubated with a cuffed tube.

All patients followed our standard clinical protocols for nutrition, sedation and weaning from mechanical ventilation.

### Data acquisition

Demographic data, co-morbidities and pediatric index of mortality-II score were recorded. Ventilator settings, sedation level, diagnosis of sepsis, diagnosis of acute kidney injury, and failure of extubation were collected daily during the study. The use of corticosteroids was defined as administration of steroids for > 24 h. The use of neuromuscular blockade or inotropics was only considered clinically relevant if administrated for more than 12 h. Extubation failure was defined as requirement for reintubation or non-invasive respiratory support within 48 h following extubation. For level of sedation, the COMFORT scale was used [12]. The data collected as part of this study were not used for clinical decisions as treating clinicians were not aware of the results.

### Ultrasound measurements

Daily ultrasound measurements were performed in 2D mode with the PHILIPS CX50 (Andover, Massachusetts, USA) using the high-frequency (15–7 MHz) linear array transducer. All measurements were performed in semi-recumbent position.

#### Diaphragm

**Fig. 1:**
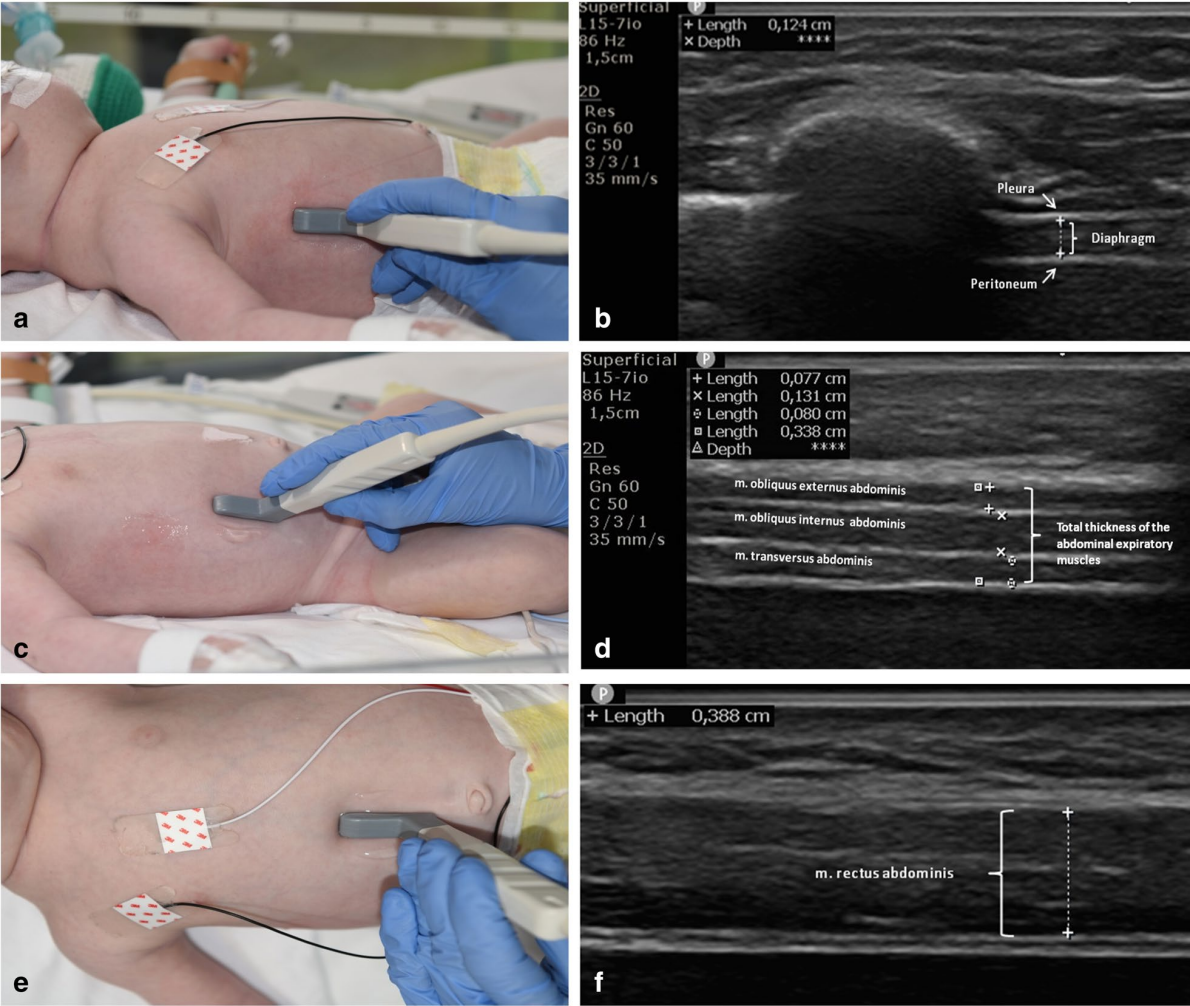
Ultrasound position and measurements of diaphragmatic thickness in the zone of apposition (**a**, **b**) of the 3 abdominal muscles (m. obliquus externus abdominis, m. obliquus internus abdominis, m. transversus abdominis), separate and together (**c**, **d**); and of the m. rectus abdominis (**e**, **f**) with a 7–15 MHz linear array transducer. Pictures were taken with the informed consent of the legal guardians

Thickness of the diaphragm was measured as described previously [13, 14]. In brief, the diaphragm was visualized placing the transducer perpendicular to the chest wall in the 9th or 10th intercostal space between the anterior and mid-axillary lines where the zone of apposition was identified. In this region, the diaphragm was visualized as a 3-layered structure: a non-echogenic central layer and two echogenic layers (the peritoneum and pleura layer) (Fig. 1a, b). Thickness of the diaphragm was measured during end-expiration (Tdi_exp_) and at end-inspiration (Tdi_insp_) in B-mode. Thickening fraction (TF_di_) was used as an estimate of diaphragm contractile activity [15–18] and was defined as: (Tdi_insp_–Tdi_exp_/Tdi_exp_) × 100% [16]. Tdi was defined as the distance between the inner line of the diaphragmatic pleura and the peritoneum [13] (Fig. 1b). All measurements were performed at the right diaphragm because of higher feasibility and reproducibility [18]. In addition, for optimal accuracy, the following steps were added. First, the location of the transducer was marked in order to increase the day-to-day measurement consistency, as the thickness of the diaphragm is heterogeneous across its surface [18, 19]. Second, three separate consecutive measurements of end-expiratory and end-inspiratory thickness were measured, and the mean recorded to minimize intra-observer variability.

#### Expiratory muscles

The three muscular layers of the abdominal wall were visualized by positioning the transducer perpendicular between the costal margin and iliac crest at the right mid-axillary or anterior axillary line (Fig. 1c). From the anterior to the posterior surface the external oblique muscle, internal oblique muscle, and transverse abdominal muscle are visualized as hypo-echoic longitudinal bands. The muscular fascia between these muscles is visible as a hyper-echoic band (Fig. 1d). To reduce intra-observer variability, three consecutive measurements of the expiratory muscles were obtained and the mean was calculated [20]. In every cycle the thickness of the three muscles (obliquus externa, obliquus interna and transversus abdominis) was measured separately, not including the intermuscular septa. In addition, these three muscles were measured together, from the inferior inner edge of the transversus abdominis to the superior inner edge of the obliquus externa, and therefore this measurement included the two intermuscular septa.

To visualize the rectus abdominis muscle the transducer was placed perpendicular 2 cm above the umbilicus and ± 2 cm lateral from the linea alba (Fig. 1e, f) [21].

The measurements of the expiratory muscles were performed at end-inspiration, during the relaxation of the abdominal muscles, and at end-expiration. Effort of the expiratory muscles was quantified using the thickening fraction: (thickness_exp_–thickness_insp_/thickness_insp_) × 100%. Measurements were performed on the right side of the body only assuming symmetry of the abdominal muscles [22]. The location of the transducer was marked on the skin in order to increase the day-to-day measurements consistency.

All ultrasound measurements were performed by a single pediatric intensivist (MIJ) with an extensive experience in muscle ultrasound.

### Data treatment and statistical analyses

Statistical analysis was performed with SPSS (version 25.0, Chicago, IL. USA) and GraphPad PRISM (version 5.03, San Diego, CA, USA). Data were tested for normal distribution and expressed as median ± interquartile range [IQR] for nonparametric variables and numbers (percentage). Wilcoxon signed rank test and Kruskal–Wallis test were used to compare continuous nonparametric variables between 2 and ≥ 2 groups, respectively. Chi-square test was used to compare for categorical variables. If in Kruskal–Wallis test the *P* value was observed to be significant, the difference in medians between groups were further assessed using Mann–Whitney U test. Statistical significance was indicated by *P* ≤ 0.05.

Pearson correlation coefficients (*r*) were used to assess relationships between changes in thickness of the different respiratory muscles and the relationships between changes in thickness of the different respiratory muscles and corresponding thickening fraction after inspection of data with a two-way scatter plot.

It is known that most profound changes in the thickness of the diaphragm occur during the first 2–4 days of mechanical ventilation [3, 23]. The study population was divided into three groups, based on > 10% decrease, ≤ 10% change or > 10% increase in diaphragm thickness, based on earlier studies [23]. This change was measured from baseline to the final measurement ≤ day 4 of mechanical ventilation (i.e., if a patient was extubated on day 3 of mechanical ventilation, the change in diaphragm thickness (%) was calculated from baseline to day 3). This distribution in three groups was also made for changes in thickness of the expiratory muscles.

Intra-observer reproducibility of ultrasound measurements was assessed by computing intraclass correlation coefficient.

## Results

### Patient’s characteristics

Demographic and clinical characteristics for patients (*N *= 35) are shown in Table 1. In two patients high-flow nasal therapy was used for up to 6 h after extubation, and in one patient non-invasive ventilation was applied < 2 h immediately after extubation. All three patients required tracheal re-intubation due to inadequate airway clearance.

**Table 1:** Patient characteristics for overall study cohort

Characteristics	*n *= 34
Age, months	5.5 (1–28.3)
Sex, female	19 (56)
Body weight, kg	6.6 (4.8–13.6)
Pediatric index of mortality 2 score, %	1.64 (0.6–5)
Admission diagnosis	
Bronchiolitis	17 (50)
Pneumonia	7 (20.6)
Upper airway obstruction	3 (8.8)
Status asthmaticus	2 (5.9)
Post-cardiac arrest	1 (2.9)
Neurological disease/trauma	3 (8.8)
Severe sepsis	1 (2.9)
Subjects with co-morbidities	11 (32.4)
Initial ventilator mode	
Controlled (PCV, PRVC, VCV)	22 (64.7)
Partial assist (PSV)	12 (35.3)
Initial ventilator settings	
V_T_, ml/kg	6.4 (5.8–7)
PEEP, cmH_2_O	5 (4–6)
FiO_2_	0.4 (0.3-0.6)
Peak-PEEP, cmH_2_O	17.5 (11.8–25.2)
Kidney failure	2 (5.9)
Inotropes (> 12 h)	3 (8.8)
Vasopressors (> 12 h)	6 (18.8)
Neuromuscular blockade (> 12 h)	3 (8.8)
Systemic corticosteroids (> 24 h)	6 (17.6)
Failed extubation	3 (8.8)
Duration of MV, hours	112 (67–159)
PICU length of stay, days	6 (4–9)
Mortality	3 (8.8)

Results are presented as median (IQR) or number (percent)

*IQR,* interquartile range; *MV,* mechanical ventilation; *PCV*, pressure control ventilation; *PRVC*, pressure regulated volume control; *VCV*, volume controlled ventilation; *PSV*, pressure support ventilation; *V*_*T*_, tidal volume; *PEEP*, positive end-expiratory pressure; *kg*, kilogram

In one patient only one ultrasound measurement could be obtained and was excluded from further analysis. Of the remaining 34 patients, 23 (68%) underwent 4 ultrasound measurements, 8 (23%) patients underwent 3 ultrasound measurements and in 3 (9%) patients 2 measurements were obtained. The major reasons for obtaining ≤ 4 ultrasound measurements were early extubation (*n* = 9), mortality (*n* = 1), and in one patient parents withdrew consent for further measurements. In total 366 ultrasounds and 4392 measurements were obtained in 34 patients.

In all patients the first ultrasound measurements were obtained < 24 h after intubation (mean 14.9 h; SD ± 8.2). Due to progressive respiratory failure, veno-venous extracorporeal membrane oxygenation was initiated in two patients.

In total 125 patients were eligible for the study (see Additional file 1) of which 34 patients were included.

### Diaphragm ultrasound measurements

**Fig. 2:**
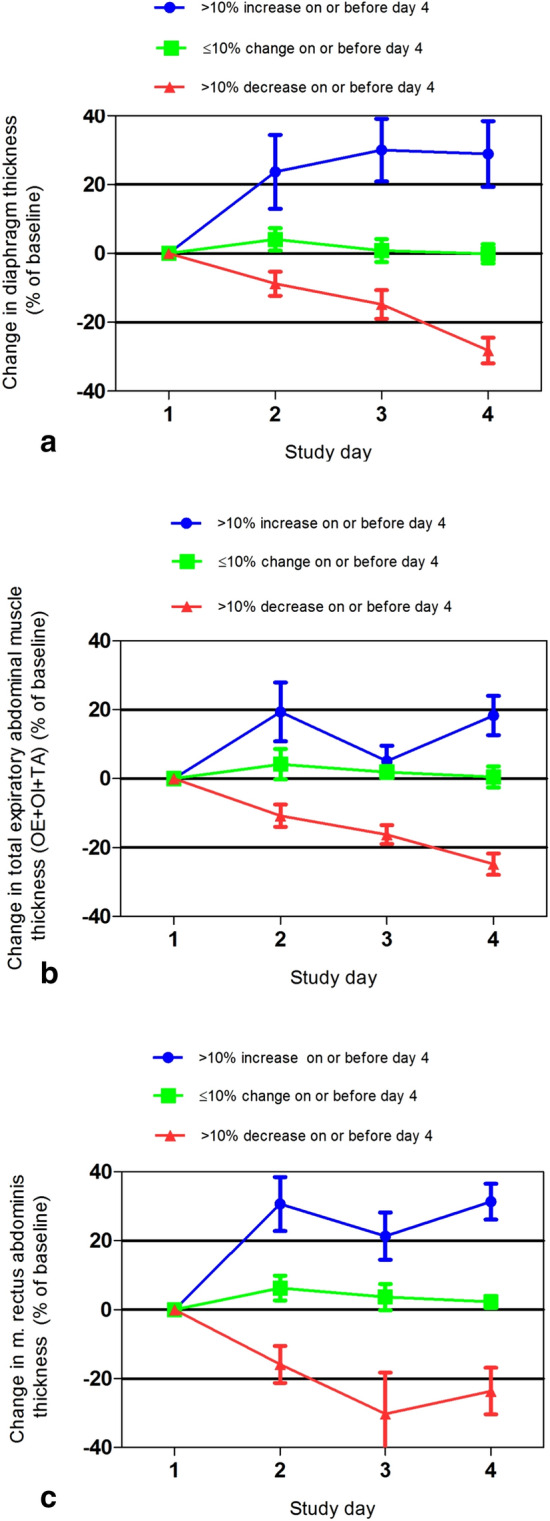
Change in thickness of the diaphragm muscle (**a**), total expiratory muscles (OE + OI + TA) (**b**), and m. rectus abdominis (**c**) over the first 4 days of mechanical ventilation. Mean and standard error of the mean are plotted for each group and study day. Thickness of the muscles was significantly different between each group for all muscles on day 4 (*P* ≤ 0.0001). *OE*, obliquus externa; *OI*, obliquus interna; *TA*, transversus abdominis

The median change in diaphragm thickness on day 4 of mechanical ventilation compared to baseline for the three predefined groups was: − 21.2% (IQR, − 38.0–− 15.8) for the > 10% decrease in diaphragm thickness group (*N *= 15), 1.5% (IQR, − 6.1–7.9) for the ≤ 10% change in diaphragm thickness group (*N *= 10) and 26.3% (IQR, 12.0–51.1) for the > 10% increase in diaphragm thickness group (*N *= 9) (Fig. 2a; *P* ≤ 0.0001). Measurements of diaphragm thickness at baseline and on day 4 of mechanical ventilation are reported in Table 2.

**Table 2:** Diaphragm thickness and thickening fraction based on changes in thickness in first 4 days of mechanical ventilation

	Overall study population (*n* = 34)	> 10% decrease (*n* = 15)	Within 10% of baseline (*n* = 10)	>10% increase (*n* = 9)	*P* value
Baseline measurements					
Thickness at end-expiration (mm)	1.5 (1–1.6)	1.6 (1.1–1.6)	1.4 (0.8–1.6)	1.1 (1.0–1.5)	0.253
Thickening fraction (%)	10.7 (7.5–16.2)	9.1 (4.9–13.0)	16.3 (11.9–23.6)	10.7 (7.3–13.2)	0.029^a^
Last measurements (day ≤ 4)					
Thickness at end-expiration (mm)	1.3 (0.9–1.6)	1.2 (0.8–1.4)	1.3 (0.9–1.7)	1.6 (1.3–1.7)	0.017^b^
Thickening fraction (%)	12.4 (5.7–16.6)	7.1 (0.9–15.6)	12.6 (9.7–15.3)	16 (11.0–21.7)	0.147
Average over first 4 days					
Thickening fraction (%)	11.4 (9.7–14.5)	9.9 (8.6–12.2)	12.8 (10.5–18.1)	12.5 (10.6–16.4)	0.051^c^

Data are presented as median (IQR)

Patients are divided into 3 subgroups according to the change in diaphragm thickness during ≤ 4 days of MV

*IQR*, interquartile range, *MV*, mechanical ventilation

^a^Between patients with > 10% decrease and ≤ 10% change in diaphragm muscle thickness, and between patients with > 10% increase and ≤ 10% change in diaphragm muscle thickness

^b^Between patients with > 10% increase and patients ≤ 10% change in diaphragm thickness

^c^Between patients with > 10% decrease and ≤ 10% change in diaphragm thickness

No significant changes in the clinical characteristics among groups were found (see Additional file 2). However, children with > 10% decrease in diaphragm thickness were ventilated with a significantly higher median tidal volume over the first 4 days and were significantly older compared to the other two groups. However, no significant correlation between age and diaphragm thickness at baseline or between age and changes in diaphragm thickness over the first 4 days of mechanical ventilation was observed (see Additional file 3).

Measurements of the thickening fraction of the diaphragm (TF_di_) are shown in Table 2. The average TF_di_ over 4 days during mechanical ventilation was significantly lower in patients with ≥ 10% decrease in diaphragm thickness compared to patients with ≤ 10% change in diaphragm thickness. However, for the entire population no significant correlation was found between the changes in diaphragm thickness during the first 4 days of mechanical ventilation and the average TF_di_ over the first 4 days of mechanical ventilation (see Additional file 4).

### Expiratory muscles measurements

The thickness of the three abdominal wall muscles together (obliquus interna, obliquus externa and transversus abdominis) for the overall study population decreased during the first 4 days of mechanical ventilation. This decrease is mainly determined by the decrease in the thickness of the obliquus interna (Table 3).

**Table 3:** Ultrasound thickness measurements of the abdominal expiratory muscles

	Baseline measurement (*n *= 34)	Last measurement (*n* = 34)	*P* Value
Total expiratory abdominal muscles (OE + OI + TA) (mm)	4.2 (3.6–5.3)	4.1 (3.5–5.3)	0.019
m. Obliquus externa (mm)	0.9 (0.8–1.1)	1.0 (0.8–1.1)	0.748
m. Obliquus interna (mm)	1.3 (0.9–1.8)	1.2 (1.0–1.7)	0.032
m. Transversus abdominis (mm)	1.2 (0.9–1.6)	1.2 (0.9–1.5)	0.067
m. Rectus abdominis (mm)	1.9 (1.5–2.3)	2.2 (1.8–2.7)	0.007

Results are presented as median (IQR)

Thickness of the total expiratory muscles includes the sum of the thickness of the OE, OI and TA. The last measurement was defined as the final measurement ≤ day 4 of mechanical ventilation

*IQR*, interquartile range; *OE*, obliquus externa; *OI*, obliquus interna; *TA*, transversus abdominis

The median change in thickness of the total expiratory muscles on day 4 of mechanical ventilation for the three groups was: − 22.8% (IQR, − 32.8–− 13.1) for the > 10% decrease in thickness group (*N* = 15), − 2.6% (IQR, − 4.3–7.4) for the ≤ 10% change in thickness group (*N *= 12), and 14.5% (IQR, 10.5–27.9) for the > 10% increase in thickness group (*N* = 7) (Fig. 2b; *P* ≤ 0.0001).

Baseline measurements for the three groups are shown in the online supplement (see Additional file 5). Although a weak correlation was found between the baseline thickness of the expiratory muscles (obliquus interna, obliquus externa and transversus abdominis together) and age (see Additional file 6), no significant differences in the clinical characteristics, especially in age, among the three groups were found (see Additional file 7).

During the first 4 days of mechanical ventilation, the thickness of m. rectus abdominis for the overall study population increased (Table 3). The median change in thickness of the m. rectus abdominis on day 4 of mechanical ventilation compared to baseline for the three predefined groups is shown in Fig. 2c.

The thickening fraction of the expiratory muscles in the first 4 days was not significantly different between the three groups (see Additional file 8).

In addition, no significant correlation was found between the changes in expiratory muscle thickness during the first 4 days of mechanical ventilation and the median thickening fraction of the corresponding muscle over the first 4 days of mechanical ventilation (see Additional file 9).

As shown in Table 4, it is remarkable that the three children failing extubation exhibit a more pronounced decrease in expiratory muscle thickness (obliquus externa, obliquus interna and transversus abdominis) on day 4 of mechanical ventilation compared to children with successful extubation, although very small sample size precludes a meaningful interpretation.

**Table 4:** Characteristics and measurements of diaphragm and expiratory muscles for patients with successful and failed extubation

	Successful extubation (*n* = 31)	Failed extubation (*n* = 3)	*P* value
Clinical characteristics			
Age, months	4.0 (1–25)	15 (13–15)	0.142
Body weight, kg	6.0 (4.3–13.5)	8.9 (8.0–8.9)	0.261
Duration of MV, h	103 (67–154)	268 (120–268)	0.073
Ventilator settings^a^			
Peak-PEEP, cmH_2_O	15 (12–20)	15 (12.7–15)	0.855
Tidal volume, ml/kg	6.6 (6.0–7.3)	6.4 (5.8–6.4)	0.524
Diaphragm			
Baseline thickness	1.4 (1.0–1.6)	1.6 (1.0–1.6)	0.693
Change in thickness (% of baseline)	10.7 (9.5–14.4)	12.5 (12.1–12.5)	0.785
Average TF_di_ (first 4 days)	− 5.96 (− 20.4–10.6)	− 12.4 (− 21.9–− 12.4)	0.237
TF_di_ before extubation	15.2 (9.6–19.1)	4.0 (0–4.0)	0.108
Expiratory muscles (OE, OI, TA)			
Baseline thickness	4.2 (3.6–5.3)	4.2 (3.5–4.2)	0.649
Change in thickness (% of baseline)	− 3.89 (− 16.6–9.5)	− 33.8 (− 43–− 33.8)	0.014
Average TF (first 4 days)	5.38 (2.8–6.8)	3.46 (2.0–3.46)	0.236
TF before extubation	6.5 (1.9–10.6)	3.5 (0–3.5)	0.395
Rectus abdominis (RA)			
Baseline thickness	1.9 (1.6–2.3)	1.6 (1.2–1.6)	0.485
Change in thickness (% of baseline)	8.12 (–0.9–22.6)	0.54 (–16.8–0.54)	0.564
Average TF (first 4 days)	2.33 (1.7–4.1)	2.5 (0.6–2.50)	0.785
TF before extubation	1.3 (0–4.2)	1.4 (0–1.4)	0.975

Results are presented as median [IQR]; successful extubation group (Q_1_–Q_3_) and failed extubation group (Q_1_–Q_2_)

*IQR*, interquartile range, *TF*, thickening fraction; *TF*_*di*_, thickening fraction diaphragm at end-inspiration

*OE*, m. obliquus externa; *OI*, m. obliquus interna; *TA*, m. transversus abdominis; *RA*, m. rectus abdominis

^a^The ventilation settings are shown as an average over the first 4 days of mechanical ventilation

### Correlation between the change in diaphragm thickness and expiratory muscles thickness

No significant correlation was found between changes in diaphragm thickness and changes of the three abdominal wall muscles together (obliquus externa, obliquus interna and transversus abdominis) and rectus abdominis (see Additional file 10). However, a weak, but significant correlation was found between the direction of change in diaphragm thickness and the obliquus externa (*r*^2^ = 0.125, *P *= 0.041) (see Additional file 10b).

Between the expiratory muscles (obliquus externa, obliquus interna and transversus abdominis), in particular between the obliquus interna and transversus abdominis, a strong and significant correlation was found between the change in muscle thickness during the first 4 days of mechanical ventilation (see Additional file 11).

### Reproducibility

The correlation coefficients of intra-observer reproducibility of the different muscles were excellent; 0.997 (0.994–0.998), 0.999 (0.999–1.000), 0.985 (0.973–0.992), 0.998 (0.996–0.999), 0.999 (0.998–0.999) for the Tdi_exp_, the total thickness of the expiratory abdominal muscles together (obliquus externa, obliquus interna and transversus abdominis), obliquus externa, obliquus interna, transversus abdominis and rectus abdominis, respectively.

## Discussion

This is the first study to evaluate the effects of critical illness on the expiratory muscles, an important component of the respiratory muscle pump, in mechanically ventilated children. In these patients, changes in thickness of the expiratory muscles are unpredictable, but develop rapidly after the initiation of mechanical ventilation. Changes in thickness of the expiratory muscles are not correlated with changes in thickness of the diaphragm, indicating different vulnerability of these muscles to various stimuli present in critically ill mechanically ventilated children. Also, we found that a decrease in thickness of the expiratory muscles was more pronounced in patients who failed extubation. Finally, we report for the first time that assessment of expiratory muscle thickness is feasible and highly reproducible in PICU patients. These observations provide new insights into the effects of critical illness on the respiratory muscle pump and may have implications for monitoring and treatment of ventilated children.

### Diaphragm

In our study, diaphragm atrophy developed in 44% of the children during the first 4 days of mechanical ventilation. This is in line with previous observations in mechanical ventilated children [3–5]. As the median tidal volume over the first 4 days in this group of children was significantly higher compared to the other two groups, ventilator over-assist could be a contributing factor in the development of diaphragm atrophy in this group. Unfortunately, esophageal pressure was not measured in the current study and therefore this conclusion remains speculative.

The thickness of the diaphragm (Tdi) (1.5 mm) at study inclusion is similar as reported by Lee [3], but slightly lower compared to Glau (2.0 mm) [5]. A possible explanation is how Tdi was defined in the latter study: outer edge of the peritoneal membrane to the outer edge of the diaphragmatic pleura, thus the peritoneum and pleura were included in thickness of the diaphragm. In our study, as has been recommended previously [13], the pleura and peritoneum were not included in thickness measurements. In our opinion, this is more appropriate when ultrasound is used to assess muscle function.

#### Thickening fraction

In our study, the average TF_di_ over 4 days of mechanical ventilation was significantly lower in the group with more than 10% decrease in diaphragm thickness compared to the patient group without change in diaphragm thickness, indicating lower breathing effort. However, for the whole cohort no significant correlation between TF_di_ and change in diaphragm thickness was found. This is in apparent contrast with the findings of Goligher et al. [23] reporting low TF_di_ with decreases in diaphragm thickness and high TF_di_ with increases in diaphragm thickness in mechanical ventilated adult patients. Interestingly, previous studies have not calculated the correlation between TF_di_ and change in muscle thickness in children [3–5]. Nevertheless, it should be noted that TF_di_ is usually assessed only once daily and as the respiratory drive may vary over the day it may be a poor estimate of respiratory muscle activity throughout the day. Therefore, presence or absence of correlations between muscle activity assessed with ultrasound and patient outcome (including muscle atrophy) should be interpreted with caution.

Additionally, TF_di_ can be used to predict weaning failure, with values > 30–36% predicting successful extubation in adults [24, 25]. Although our study was not designed to predict weaning failure, it is noteworthy that 91, 2% of the children were successful extubated with a median TF_di_ of 15.2%. The relatively low values of TF_di_, especially compared to adults, at the time of successful extubation in children have been reported previously [3, 5]. Explanation for the lower TF_di_ in children at time of successful extubation, might be higher levels of sedation or higher levels of respiratory support prior to extubation compared to adults. Furthermore, the TF_di_ threshold predictive of successful extubation may be lower in the children compared to adults.

### Expiratory muscles

This study is the first to report changes in thickness of the expiratory abdominal muscles in critically ill ventilated patients.

The larger decrease in muscle thickness for the expiratory abdominal wall muscles (obliquus externa, obliquus interna, transversus abdominis) compared to the rectus abdominis muscle is incompletely understood, but may be explained by the differences in anatomy and function. The most prominent function of the rectus abdominus muscle is flexion of the spine, while activation has only limited effects on intraabdominal pressure. On the other hand, bilateral activation of the oblique abdominal muscles and transverse abdominal muscle during expiration will increase intraabdominal pressure and pleural pressure which assist lung deflation. In addition to assisting expiration, these muscles play a role in coughing and airway clearance preventing the development of atelectasis [26–29].

#### Thickening fraction

No significant difference in the thickening fraction of the expiratory muscles between the three subgroups defined by changes in muscle thickness was found, which suggests that muscle activity plays a limited role in changes of muscle thickness in the acute phase of critical illness in children. It should, however, be noted that the thickening fraction was low in all groups, with a median thickening fraction between 2.4 and 6.1% for the different muscles for the whole cohort during the first 4 days of ventilation.

Thickening of the abdominal muscles, in particular the m. transversus abdominis and obliquus interna, assessed by ultrasound can be used to quantify muscle activity [30–32]. However, the correlation between muscles thickening and muscles activity seems to be depended on the type of activity of these muscles, as has been measured previously using electromyography (EMG) [33]. For instance, a good correlation between thickening of the obliquus externa and EMG was found during isometric trunk rotation, but less in abdominal hollowing movements [31, 32]. Until now no studies have investigated the correlation between expiratory muscles thickening and EMG during mechanical ventilation. Further research is necessary to investigate if thickening of the expiratory abdominal muscles measured with ultrasound in mechanical ventilated children correlates with EMG.

#### Correlation of changes in muscles thickness between the expiratory muscles

Our data report a strong and significant correlation in the change in thickness of the four abdominal wall muscles during the first 4 days of mechanical ventilation, especially between the transverse abdominis and obliquus interna. A possible explanation for this correlation may lie in the hierarchy of recruitment of these muscles during expiration; the transverse abdominis is the most active muscle during expiration directly followed by the obliquus interna and finally by the obliquus externa and rectus abdominis [21, 27].

### Correlation of changes in diaphragm thickness and changes in expiratory muscles thickness

Although we report that changes in thickness of both the diaphragm and expiratory muscles develop during critical illness, the within a subject change in thickness of these muscles is very different. Thus despite the fact that these muscles within a single patient are exposed to the same level of systemic inflammation, oxidative stress [34, 35], drugs, toxins and so on these respiratory muscles exhibit different patterns of change in thickness. Notably, the contraction pattern of these muscles may be different, suggesting that changes in thickness are largely driven by differences in contractile activity. Furthermore, despite both muscles being skeletal muscles, there is a variation in fiber type composition between the diaphragm and expiratory abdominal wall muscles with more type I fibers in the latter muscles compared to the diaphragm [36, 37].

Our findings have important implications. Foremost, to obtain insight in the effects of critical illness and/or mechanical ventilation on the respiratory muscle pump, it is insufficient to only investigate the effects on the diaphragm. Each individual muscle should be assessed to appreciate the impact of critical illness on the respiratory muscle pump.

### Strengths and limitations

Strengths of the current study include that ultrasound measurements were performed by a single experienced investigator, reducing variability. In addition, whereas previous studies were restricted to the diaphragm, we evaluated several respiratory muscles providing novel insights in the respiratory muscle pump in critically ill children.

However, some limitations of our study should be acknowledged. First, our study population represents a high number of cases with bronchiolitis which possibly limits generalizability of the data. As bronchiolitis has a relative low disease severity score, the severity of changes in the expiratory and inspiratory muscles thickness may be more pronounced in patients with a higher disease severity score. Second, as inclusion was dependent on the scientist and availability of the ultrasound device not all eligible patients could be included in the study. For this reason selection bias cannot be ruled out completely. Third, due to the relatively small sample size correlations between the clinical characteristics and outcome measurements should be interpreted with caution and are merely hypothesis generating. In addition, our study was not designed nor powered to study the effects of changes in diaphragm and expiratory muscle thickness and contractility on difficulty to wean or success of extubation. For this reason we do not suggest causality between expiratory muscle atrophy and extubation failure. Future multicenter studies with larger sample size should be conducted to address this important topic. Fourth, no reference values according to different age groups for diaphragm thickness, TF_di_, or expiratory muscle thickness have been established in children. For this reason, it is not possible to determine if baseline measurements of the enrolled children were already decreased, as has been described in adults [38]. Fifth, expiratory muscle strength was not measured. Therefore, we cannot conclude that the decrease in expiratory muscles thickness in children with extubation failure resulted in muscle weakness. Several studies have reported the development of expiratory muscle weakness in critically ill mechanical ventilated patients [39, 40]. For measuring the strength of the expiratory muscles various techniques can be used [39, 41–44]. For example, the rise in gastric pressure during tidal forced expiration or by volitional tests of the expiratory muscles by measuring maximal expiratory pressures (MEP) or peak cough flow (PCF). However, as the first mentioned is an invasive technique and as MEP and PCF measurements require a voluntary patient effort, this is not attractive and partly impossible in most of the pediatric intensive care population. In future studies, quantification of expiratory muscle function is advisable although it is challenging to obtain reliable data in children.

## Conclusion

Changes in thickness of the expiratory abdominal muscles develop rapidly after the initiation of mechanical ventilation in critically ill children. Interestingly, changes in thickness of the expiratory muscles are not correlated with changes in diaphragm muscle thickness. These data demonstrate that distinct components of the respiratory muscle pump respond differently to mechanical ventilation and critical illness. These observations provide unique insights in the effects of critical illness on the respiratory muscle pump that may have implications for monitoring and treatment of ventilated children.

## Supplementary information

**Additional file 1:** Patient characteristics of patients eligible for the study. Results are presented as median [IQR] or number (percent). *IQR *= interquartile range.

**Additional file 2:** Patient characteristics subdivided based on changes in diaphragm thickness in the first 4 days of mechanical ventilation. Results are presented as median [IQR] or number (percent). *IQR* = interquartile range; *MV* = mechanical ventilation; *PCV* = pressure control ventilation; *PRVC*= pressure regulated volume control; *VCV* = volume controlled ventilation; *PSV* = pressure support ventilation; V_T_ = tidal volume; *PEEP* = positive end-expiratory pressure; *kg* = kilogram.

**Additional file 3:** Correlations between diaphragm thickness and age. Correlation between (a) the diaphragm thickness at baseline and age (*r* 2 = 0.032, *P* = 0.312) and between (b) the change in diaphragm thickness over the first 4 days of mechanical ventilation and age (*r* 2 = 0.033, *P* = 0.308). A regression line is indicated by the solid line.

**Additional file 4:** Correlation of the average diaphragm thickening fraction over the first 4 days of mechanical ventilation and the mean change in diaphragm thickness over the first 4 days of mechanical ventilation (*r* 2 = 0.030, *P* = 0.327). A regression line is indicated by the solid line.

**Additional file 5:** Ultrasound measurements (mm) of the expiratory abdominal muscles. The number of patients with changes in thickness of >10% decrease, within 10% of baseline value or with >10% increase in thickness for the different muscles’ groups were as follow: 15, 12, 7 (total expiratory muscles); 12, 9, 13 (OE), 16, 12, 6 (OI); 17, 6, 11 (TA); 4, 15, 15 (RA). *OE* = m. obliquus externa, *OI* = m. obliquus interna, *TA* = m. transversus abdominis, *RA* = m. rectus abdominis. ^a^Between patients with >10% increase and >10% decrease in the total expiratory muscles thickness, and between patients with >10% increase and ≤10% change in the total expiratory muscles thickness. ^b^Between patients with >10% increase and >10% decrease in the obliquus externa muscle thickness, and between patients with >10% increase and ≤10% change in the obliquus externa muscle thickness. ^c^ Between patients with >10% increase and >10% decrease in the transverse abdominal muscle thickness, and between patients with >10% decrease and ≤10% change in the transverse abdominal muscle thickness. ^d^ Between patients with >10% increase and ≤10% change in the rectus abdominal muscle thickness.

**Additional file 6:** Correlation between the total expiratory muscle thickness and age. Correlation between (a) the total expiratory muscle thickness at baseline and age (*r* 2 = 0.391, *P* ≤ 0.001) and between (b) the change in total expiratory muscle thickness over the first 4 days of mechanical ventilation and age (*r* 2 = 0.023, *P* = 0.390). A regression line is indicated by the solid line. *OE* = m. obliquus externa, *OI* = m. obliquus interna, *TA* = m. transversus abdominis.

**Additional file 7:** Characteristics subdivided based on changes in total expiratory muscle thickness within the first 4 days of mechanical ventilation. The total expiratory muscles include the thickness of the m. obliquus externa, m. obliquus interna and m. transversus abdominis together. Results are presented as median [IQR] or number (percent). *IQR* = interquartile range; *MV* = mechanical ventilation; *PCV* = pressure control ventilation; *PRVC*= pressure regulated volume control; *VCV* = volume controlled ventilation; *PSV* = pressure support ventilation; V_T_ = tidal volume; *PEEP* = positive end-expiratory pressure; *kg* = kilogram.

**Additional file 8:** Thickening fraction at end-expiration of the expiratory abdominal muscles is expressed in percentage (%). Patients are divided in 3 groups according to the change in expiratory muscles thickness during the first 4 days of MV. The number of patients with changes in thickness of >10% decrease, ≤10% change or with >10% increase in thickness for the different muscles’ groups were as follow: 15, 12, 7 (total expiratory muscles); 12, 9, 13 (OE), 16, 12, 6 (OI); 17, 6, 11 (TA); 4, 15, 15 (RA). *OE* = m. obliquus externa, *OI* = m. obliquus interna, *TA* = m. transversus abdominis, *RA* = m. rectus abdominis, *MV* = mechanical ventilation.

**Additional file 9:** Correlation between changes in expiratory muscle thickness and average thickening fraction of the corresponding muscle over the first 4 days of mechanical ventilation. *OE* = m. obliquus externa, *OI* = m. obliquus interna, *TA* = m. transversus abdominis.

**Additional file 10 :** Correlation between the direction of change in diaphragm thickness and the direction of change in expiratory muscles thickness. *OE* = m. obliquus externa, *OI* = m. obliquus interna, *TA* = m. transversus abdominis, *RA* = m. rectus abdominis.

**Additional file 11:** Correlation between the changes in muscles thickness between the various expiratory muscles during the first 4 days of mechanical ventilation. *OE* = m. obliquus externa, *OI* = m. obliquus interna, *TA* = m. transverse abdominis, *RA* = m. rectus abdominis.

## Data Availability

The datasets analyzed during the current study are available from the corresponding author on reasonable request.

## Abbreviations

EMG: Electromyography
IQR: Interquartile range
Kg: Kilogram
MV: Mechanical ventilation
OE: m. Obliquus externa
OI: m. Obliquus interna
PCV: Pressure control ventilation
PEEP: Positive end-expiratory pressure
PICU: Pediatric intensive care unit
PRVC: Pressure regulated volume control
PSV: Pressure support ventilation
RA: m. Rectus abdominis
TA: m. Transversus abdominis
Tdi: Thickness of the diaphragm
TF: Thickening fraction
TF_di_: Thickening fraction of the diaphragm
*V*_T_: Tidal volume
VCV: Volume controlled ventilation
