# Supplementary material for: The impact of critical illness on the expiratory muscles and the diaphragm assessed by ultrasound in mechanical ventilated children
Source: Ann Intensive Care. 2020 Aug 27;10:115. doi: 10.1186/s13613-020-00731-2 (PMC7450159; doi:10.1186/s13613-020-00731-2)
Supplement: Supplementary file 1 — Additional file 1: Patient characteristics of patients eligible for the study. Results are presented as median [IQR] or number (percent). IQR = interquartile range. [file 13613_2020_731_MOESM1_ESM.pdf]

Additional file 1

Patient characteristics of patients eligible for the study

Characteristics	*n*=125
Age, yr	1.26 (0.22-8.8)
Sex, female	62 (49.6)
Pediatric Index of Mortality 2 score, %	4.7 (1.7-8.0)
Admission diagnosis	
Bronchiolitis	42 (33.6)
Pneumonia	20 (16.0)
Upper airway obstruction	19 (15.2)
Status asthmatic	5 (4.0)
Post cardiac arrest	6 (4.8)
Neurological disease/trauma	23 (18.4)
Severe sepsis	6 (4.8)
Other	4 (3.2)
PICU length of stay, days	5 (4-8)
Mortality	9 (7.2)
