# Supplementary material for: The impact of critical illness on the expiratory muscles and the diaphragm assessed by ultrasound in mechanical ventilated children
Source: Ann Intensive Care. 2020 Aug 27;10:115. doi: 10.1186/s13613-020-00731-2 (PMC7450159; doi:10.1186/s13613-020-00731-2)
Supplement: Supplementary file 2 — Additional file 2: Patient characteristics subdivided based on changes in diaphragm thickness in the first 4 days of mechanical ventilation. Results are presented as median [IQR] or number (percent). IQR = interquartile range; MV = mechanical ventilation; PCV = pressure control ventilation; PRVC= pressure regulated volume control; VCV = volume controlled ventilation; PSV = pressure support ventilation; VT = tidal volume; PEEP = positive end-expiratory pressure; kg = kilogram. [file 13613_2020_731_MOESM2_ESM.pdf]

Additional file 2

Patient characteristics subdivided based on changes in diaphragm thickness < first 4 days of mechanical ventilation

Characteristics	>10% decrease (n =15)	≤10% change (n = 10)	>10% increase (n = 9)	*P* Value
Age. months	23 (6-68)	2 (1-5.5)	1 (1-8)	0.013
Sex, female	9 (6)	5 (50)	5 (55.6)	0.885
Body weight, kg	10 (6.4-27.0)	5.7 (4.2-9.7)	5.1 (4.3-7.5)	0.071
Pediatric Index of Mortality 2 score, %	2.31 (0.6-5.8)	1.25 (0.9-3.7)	1 (0.4-3)	0.515
Admission diagnosis				0.600
Bronchiolitis	5 (33.3)	6 (60)	6 (66.7)	
Pneumonia	3 (20)	2 (20)	2 (22.2)	
Upper airway obstruction	2 (13.3)	1 (10)	0	
Status asthmatic	2 (13.3)	0	0	
Post cardiac arrest	1 (6.7)	0	0	
Neurological disease/trauma	2 (13.3)	0	1 (11.1)	
Severe sepsis	0	1 (10)	0	
Subjects with co morbidities	6 (40)	3 (30)	2 (22.2)	0.654
Initial ventilator mode				0.183
Controlled (PCV, PRVC, VCV)	9 (60)	5 (50)	8 (88.9)	
Partial assist (PSV)	6 (40)	5 (50)	1 (11.1)	
Initial ventilator settings				
V_T, ml/kg	7.0 (5.9-8)	6.4 (5.5-6.8)	6.1 (5.8-6.5)	0.249
PEEP, cmH_2O	5 (2-7)	5 (3.75-6.0)	6 (4-7)	0.743
FiO_2	0.4 (0.3-0.5)	0.4 (0.3-0.6)	0.4 (0.4-0.7)	0.700
Peak - PEEP, cmH_2O	15 (10-27)	21.5 (11.5-6.3)	18 (13.5-21.5)	0.757
Ventilator settings (average first 4 days)				
V_T, ml/kg	7.1 (6.4-7.8)	6.1 (5.3-6.7)	6.2 (5.8-6.4)	0.012
PEEP, cmH_2O	5 (3.3-6.7)	5.3 (4-5.7)	6 (4.7-7.2)	0.410
FiO_2	0.4 (0.3-0.5)	0.4 (0.3-0.5)	0.4 (0.4-0.7)	0.679
Peak – PEEP, cmH_2O	15 (10.8-18)	17.5 (11.6-23.2)	18 (13.5-20.3)	0.629
Kidney failure	0	1 (10)	1 (11.1)	0.430
Inotropes (>12 hrs)	2 (13.3)	1 (10)	0	0.531
Vasopressors (>12 hrs)	4 (26.7)	1 (10)	1 (11.1)	0.471
Neuromuscular blockade (>12 hrs)	3 (20)	0	0	0.124
Systemic corticosteroids (>24 hrs)	3 (20)	2 (20)	1 (11.1)	0.835
Failed extubation	2 (13.3)	0	1 (11.1)	0.495
Duration of MV, hours	148 (94-268)	69.5 (65-112.5)	120 (76-157.5)	0.080
PICU length of stay, days	7 (4-16)	5 (4.8-7.5)	7 (4-8.5)	0.771
Mortality	2 (13.3)	0	1 (11.1)	0.495
