# Supplementary figures and images for: The impact of critical illness on the expiratory muscles and the diaphragm assessed by ultrasound in mechanical ventilated children
Source: Ann Intensive Care. 2020 Aug 27;10:115. doi: 10.1186/s13613-020-00731-2 (PMC7450159; doi:10.1186/s13613-020-00731-2)

Additional file 3

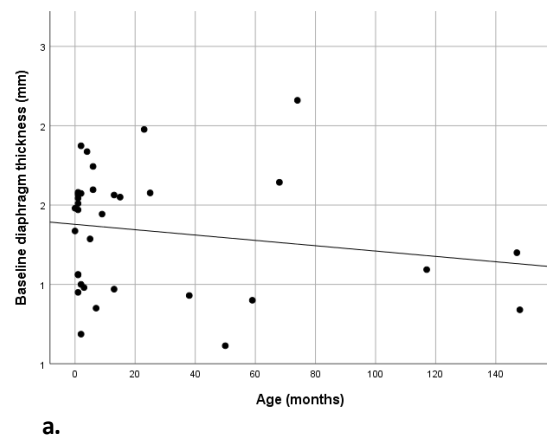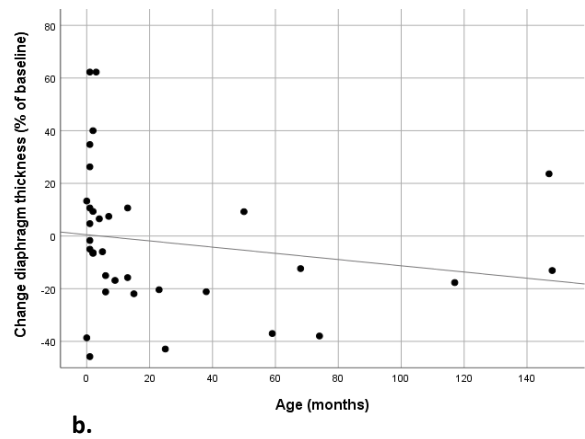

Supplement: Supplementary file 3 — Additional file 3: Correlations between diaphragm thickness and age. Correlation between (a) the diaphragm thickness at baseline and age (r 2 = 0.032, P = 0.312) and between (b) the change in diaphragm thickness over the first 4 days of mechanical ventilation and age (r 2 = 0.033, P = 0.308). A regression line is indicated by the solid line. [file 13613_2020_731_MOESM3_ESM.pdf]

#### Additional file 4

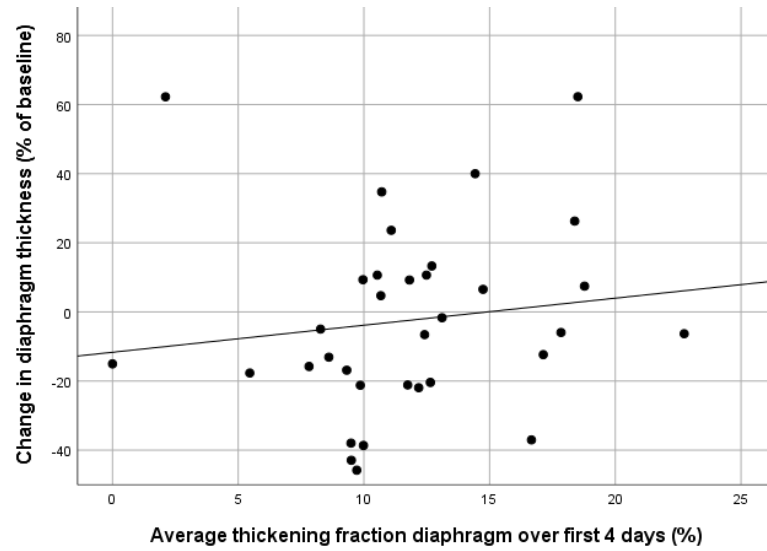

Supplement: Supplementary file 4 — Additional file 4: Correlation of the average diaphragm thickening fraction over the first 4 days of mechanical ventilation and the mean change in diaphragm thickness over the first 4 days of mechanical ventilation (r 2 = 0.030, P = 0.327). A regression line is indicated by the solid line. [file 13613_2020_731_MOESM4_ESM.pdf]

Additional file 6

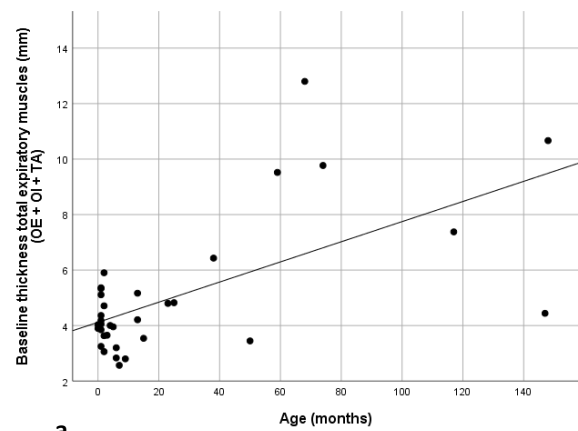

a.

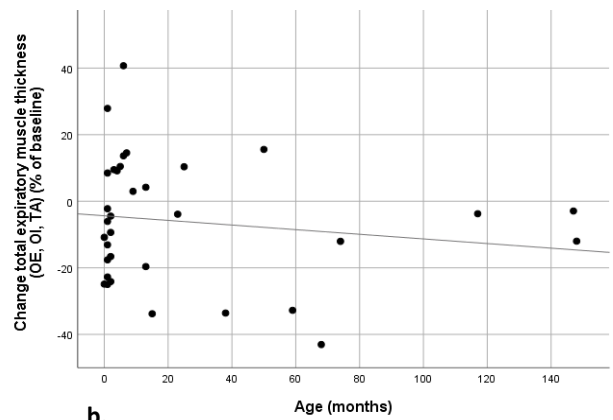

b.

Supplement: Supplementary file 6 — Additional file 6: Correlation between the total expiratory muscle thickness and age. Correlation between (a) the total expiratory muscle thickness at baseline and age (r 2 = 0.391, P ≤ 0.001) and between (b) the change in total expiratory muscle thickness over the first 4 days of mechanical ventilation and age (r 2 = 0.023, P = 0.390). A regression line is indicated by the solid line. OE = m. obliquus externa, OI = m. obliquus interna, TA = m. transversus abdominis. [file 13613_2020_731_MOESM6_ESM.pdf]

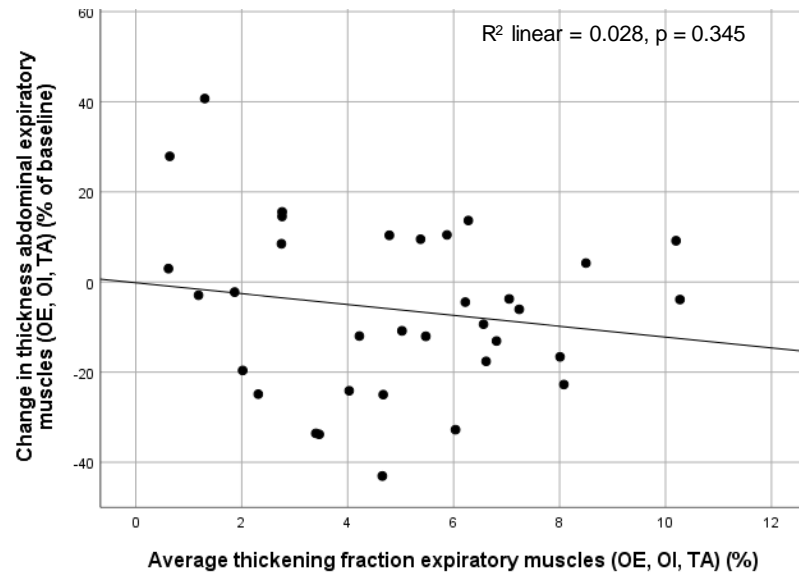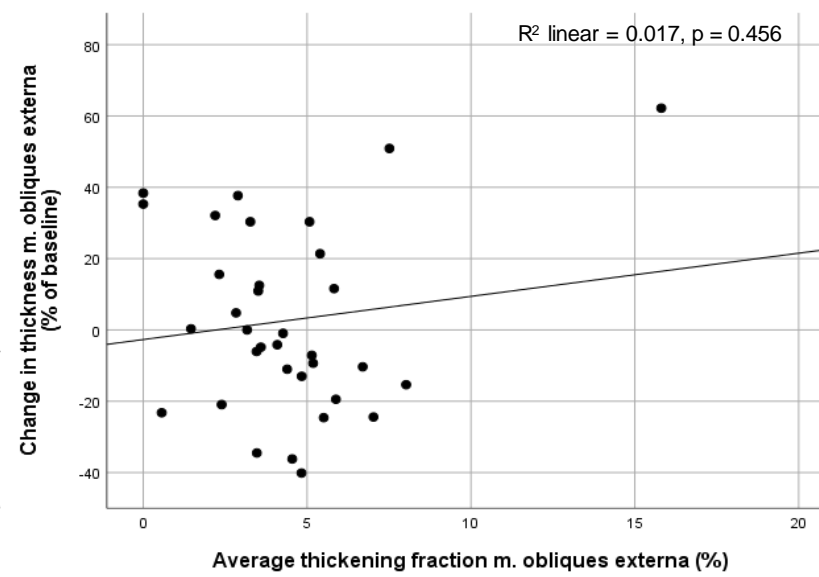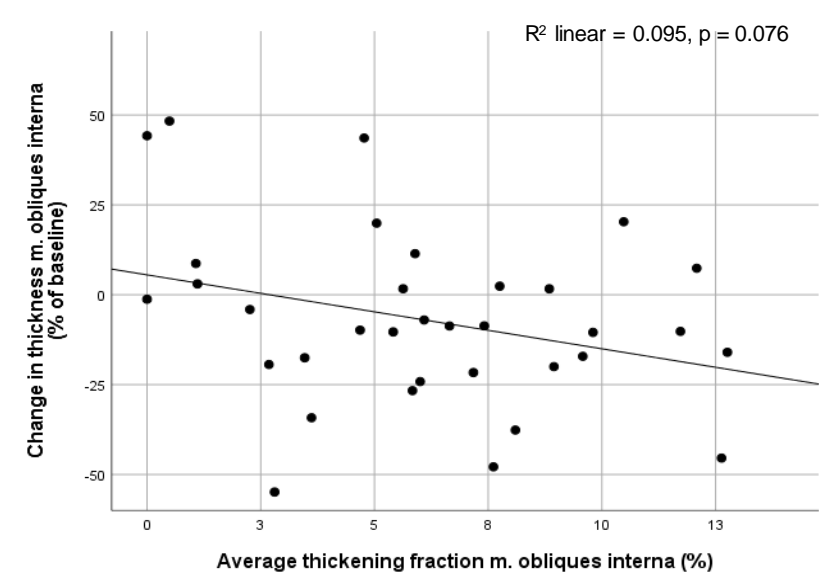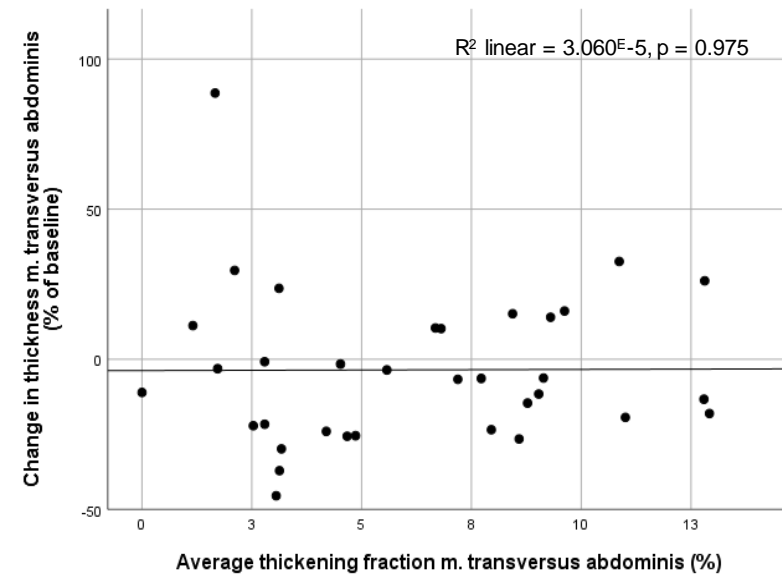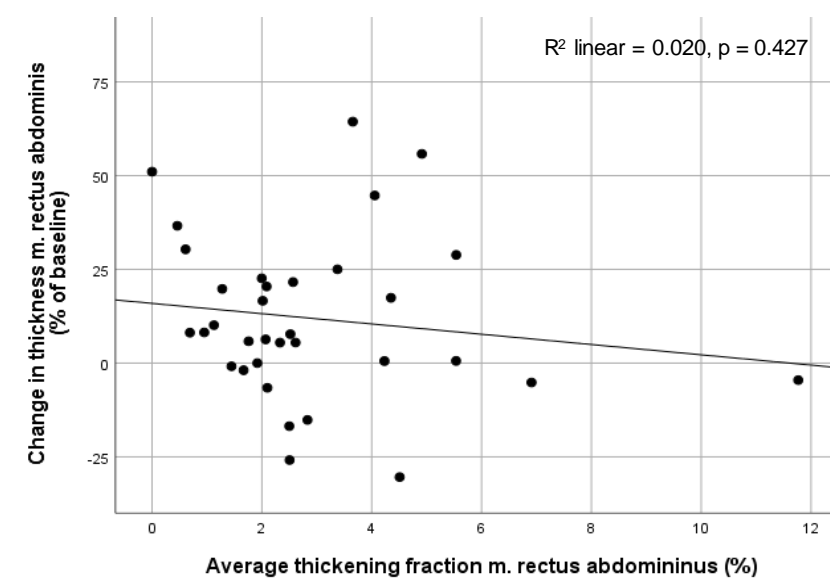

Supplement: Supplementary file 9 — Additional file 9: Correlation between changes in expiratory muscle thickness and average thickening fraction of the corresponding muscle over the first 4 days of mechanical ventilation. OE = m. obliquus externa, OI = m. obliquus interna, TA = m. transversus abdominis. [file 13613_2020_731_MOESM9_ESM.pdf]

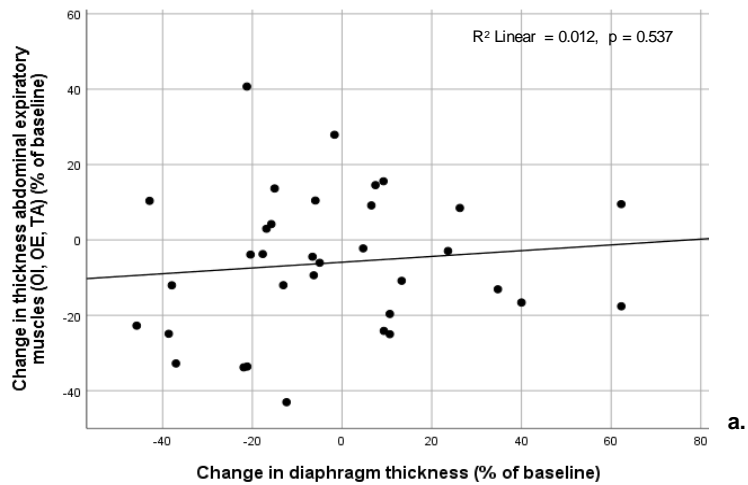

a.

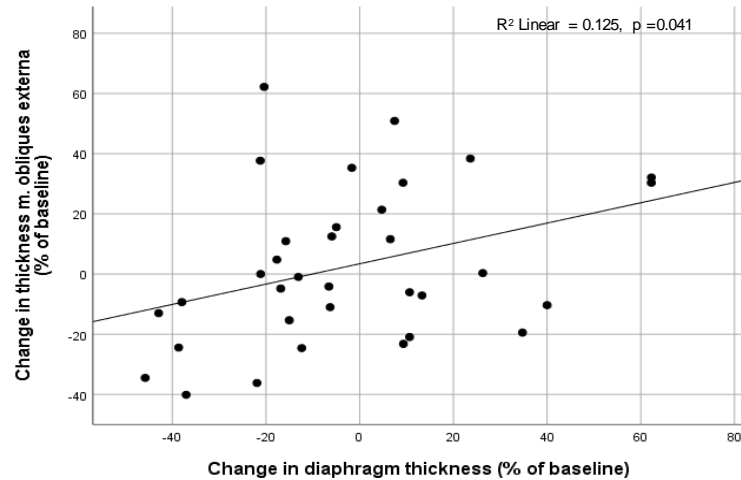

b.

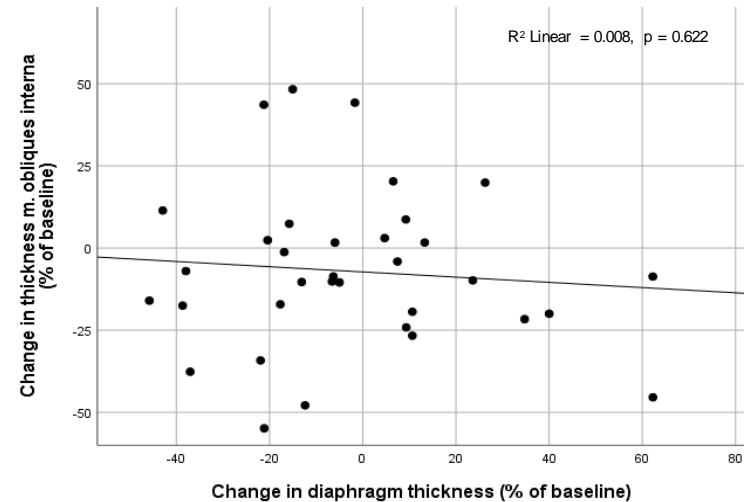

c.

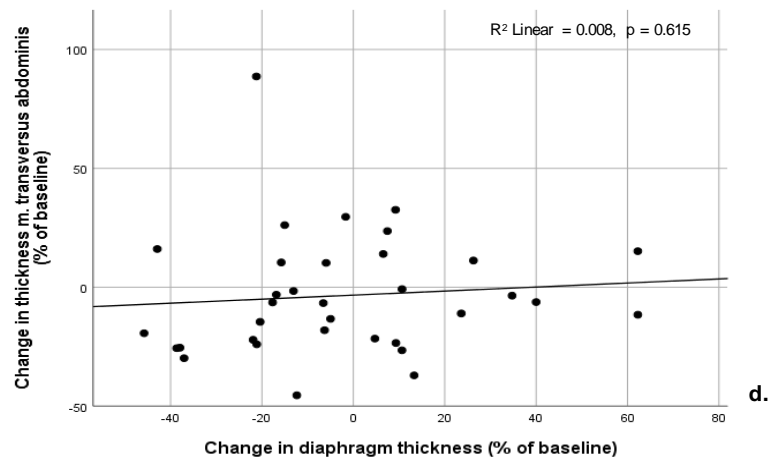

d.

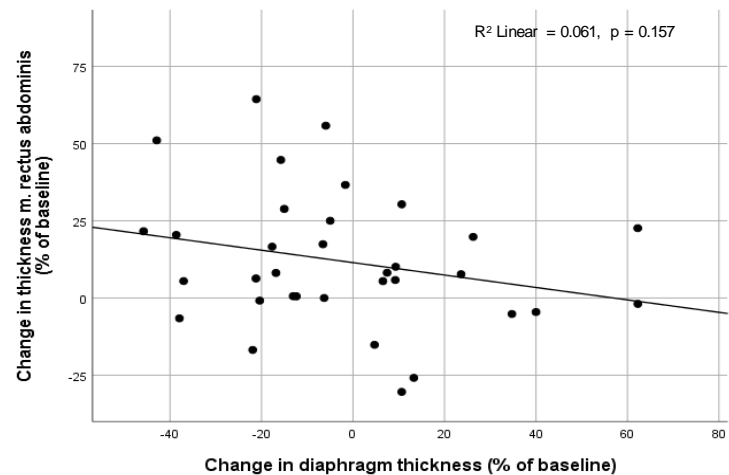

e.

Supplement: Supplementary file 10 — Additional file 10 : Correlation between the direction of change in diaphragm thickness and the direction of change in expiratory muscles thickness. OE = m. obliquus externa, OI = m. obliquus interna, TA = m. transversus abdominis, RA = m. rectus abdominis. [file 13613_2020_731_MOESM10_ESM.pdf]

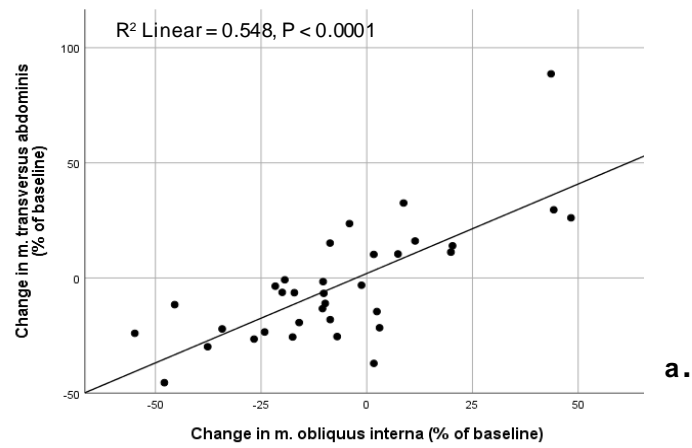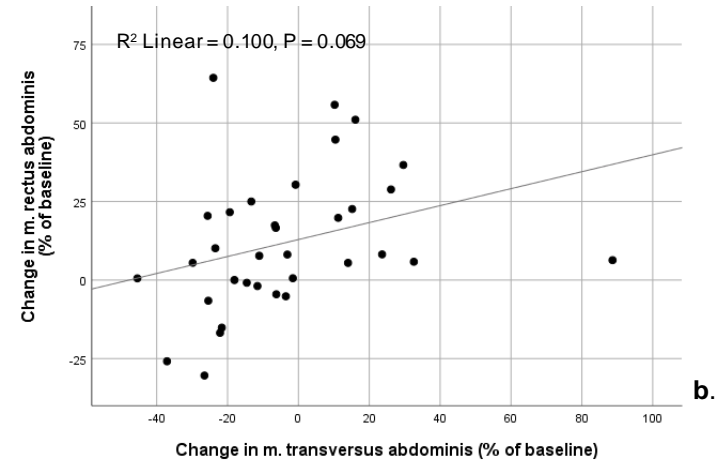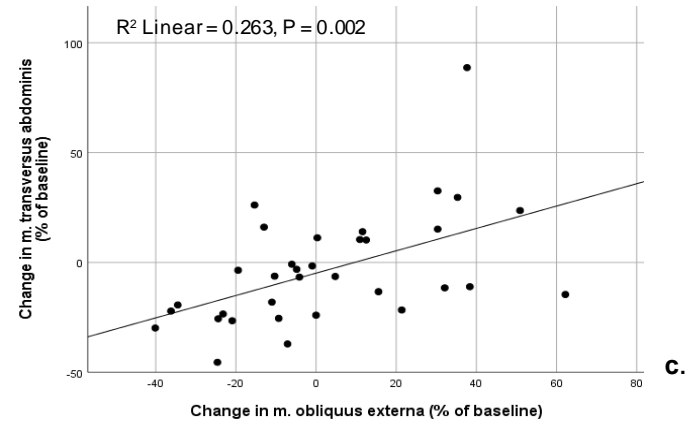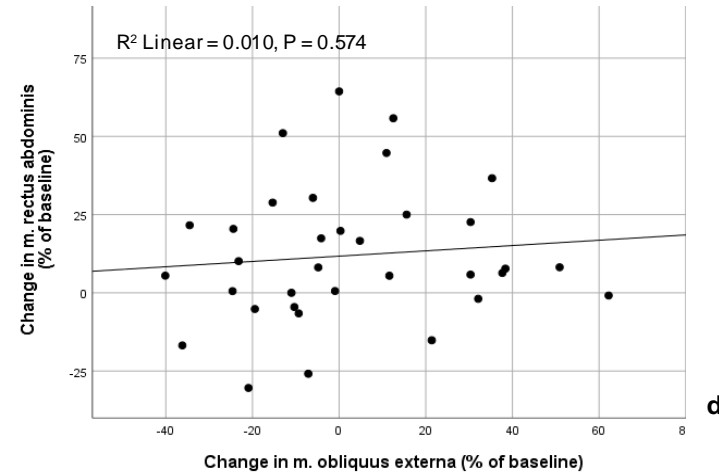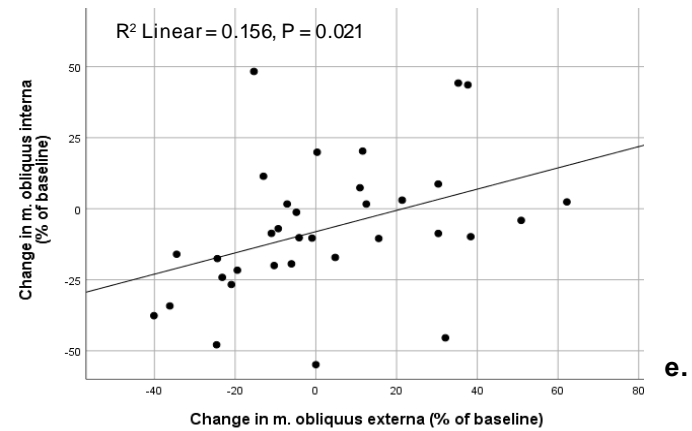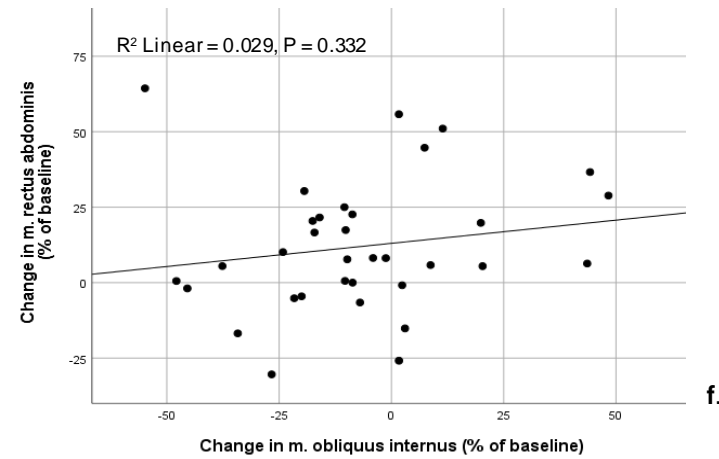

Supplement: Supplementary file 11 — Additional file 11: Correlation between the changes in muscles thickness between the various expiratory muscles during the first 4 days of mechanical ventilation. OE = m. obliquus externa, OI = m. obliquus interna, TA = m. transverse abdominis, RA = m. rectus abdominis. [file 13613_2020_731_MOESM11_ESM.pdf]
