# Supplementary material for: The impact of critical illness on the expiratory muscles and the diaphragm assessed by ultrasound in mechanical ventilated children
Source: Ann Intensive Care. 2020 Aug 27;10:115. doi: 10.1186/s13613-020-00731-2 (PMC7450159; doi:10.1186/s13613-020-00731-2)
Supplement: Supplementary file 5 — Additional file 5: Ultrasound measurements (mm) of the expiratory abdominal muscles. The number of patients with changes in thickness of >10% decrease, within 10% of baseline value or with >10% increase in thickness for the different muscles’ groups were as follow: 15, 12, 7 (total expiratory muscles); 12, 9, 13 (OE), 16, 12, 6 (OI); 17, 6, 11 (TA); 4, 15, 15 (RA). OE = m. obliquus externa, OI = m. obliquus interna, TA = m. transversus abdominis, RA = m. rectus abdominis. aBetween patients with >10% increase and >10% decrease in the total expiratory muscles thickness, and between patients with >10% increase and ≤10% change in the total expiratory muscles thickness. bBetween patients with >10% increase and >10% decrease in the obliquus externa muscle thickness, and between patients with >10% increase and ≤10% change in the obliquus externa muscle thickness. c Between patients with >10% increase and >10% decrease in the transverse abdominal muscle thickness, and between patients with >10% decrease and ≤10% change in the transverse abdominal muscle thickness. d Between patients with >10% increase and ≤10% change in the rectus abdominal muscle thickness. [file 13613_2020_731_MOESM5_ESM.pdf]

Additional file 5

Expiratory muscle thickness for study cohort and subdivided based on changes in expiratory muscle thickness in first 4 days

	Overall study population (*n* = 34)	>10% decrease	≤ 10% change	>10% increase	*P* Value
Baseline measurements					
Total expiratory muscles (OE + OI + TA)	4.2 (3.6-5.3)	4.7 (4.0-9.5)	4.4 (3.7-5.3)	3.3 (2.8-4.0)	0.013[a]
m. Obliquus externa	0.9 (0.8-1.1)	1.1 (0.9-1.4)	1.1 (0.9-1.8)	0.8 (0.7-0.8)	0.001[b]
m. Obliquus interna	1.3 (0.9-1.8)	1.4 (1.1-2.1)	1.3 (1.2-1.8)	0.9 (0.8-1.4)	0.272
m. Transverse abdominis	1.2 (0.9-1.6)	1.4 (1.1-2.1)	1.0 (0.8-1.4)	0.9 (0.9-1.3)	0.012[c]
m. Rectus abdominis	1.9 (1.5-2.3)	2.3 (1.5-3.7)	2.3 (1.9-2.5)	1.6 (1.5-1.9)	0.009[d]
Last measurements					
Total expiratory muscles (EO + OI + TA)	4.1 (3.5-5.3)	3.6 (3.2-6.4)	4.5 (3.7-5.3)	4.0 (3.6-4.4)	0.372
m. Obliquus externa	1.0 (0.8-1.1)	0.9 (0.7-1.1)	1.1 (0.8-1.8)	1.0 (0.9-1.1)	0.251
m. Obliquus interna	1.2 (1.0-1.7)	1.0 (0.9-1.3)	1.4 (1.1-1.9)	1.3 (1.1-1.7)	0.175
m. Transverse abdominis	1.2 (0.9-1.5)	1.2 (0.9-1.6)	1.0 (0.8-1.3)	1.2 (1.1-1.5)	0.237
m. Rectus abdominis	2.2 (1.8-2.7)	1.8 (1.2-2.7)	2.3 (1.8-2.7)	2.1 (1.8-2.9)	0.354
