# Supplementary material for: The impact of critical illness on the expiratory muscles and the diaphragm assessed by ultrasound in mechanical ventilated children
Source: Ann Intensive Care. 2020 Aug 27;10:115. doi: 10.1186/s13613-020-00731-2 (PMC7450159; doi:10.1186/s13613-020-00731-2)
Supplement: Supplementary file 7 — Additional file 7: Characteristics subdivided based on changes in total expiratory muscle thickness within the first 4 days of mechanical ventilation. The total expiratory muscles include the thickness of the m. obliquus externa, m. obliquus interna and m. transversus abdominis together. Results are presented as median [IQR] or number (percent). IQR = interquartile range; MV = mechanical ventilation; PCV = pressure control ventilation; PRVC= pressure regulated volume control; VCV = volume controlled ventilation; PSV = pressure support ventilation; VT = tidal volume; PEEP = positive end-expiratory pressure; kg = kilogram. [file 13613_2020_731_MOESM7_ESM.pdf]

Additional file 7

Characteristics subdivided based on changes in total expiratory muscle thickness within first 4 days of ventilation

Characteristics	>10% decrease (*n* =15)	≤10% change (*n* = 12)	>10% increase (*n* = 7)	*P* Value
Age. months	2 (1-59)	3.5 (1.3-20.5)	6 (5-25)	0.863
Sex, female	8 (53)	8 (67)	3 (43)	0.581
Body weight, kg	5.8 (4.3-17.8)	7.4 (4.6-13)	6.4 (6-11)	0.880
Pediatric Index of Mortality 2 score, %	1.0 (0.6-3.2)	1.6 (0.4-6.6)	3.3 (0.7-23.9)	0.361
Admission diagnosis				0.214
Bronchiolitis	7 (46.7)	8(66.7)	2(42.8)	
Pneumonia	2 (13.3)	2(16.7)	3(28.6)	
Upper airway obstruction	2 (13.3)	1(8.3)	0	
Status asthmatic	1 (6.7)	0	1(14.3)	
Post cardiac arrest	0	1(8.3)	0	
Neurological disease/trauma	3 (20)	0	0	
Severe sepsis	0	0	1(14.3)	
Subjects with co morbidities	5 (33.3)	4 (33.3)	2 (28.6)	0.972
Initial ventilator mode				0.645
Controlled (PCV, PRVC, VCV)	11 (73)	7 (58)	4(57)	
Partial assist (PSV)	4 (27)	5 (42)	3 (43)	
Initial ventilator settings				
V_T , ml/kg	6.3 (5.6-6.9)	6.3 (5.7-7.0)	6.8 (6.3-8.1)	0.259
PEEP, cmH_2O	5 (4-6)	5 (3.3-5.0)	6 (5-10)	0.087
FiO_2	0.4 (0.3-0.6)	0.4 (0.3-0.5)	0.6 (0.4-0.7)	0.136
Peak - PEEP, cmH_2O	15 (11-19)	22.5 (10.5-26.7)	18 (17-25)	0.569
Ventilator settings (average first 4 days)				
V_T , ml/kg	6.8 (6.0-7.8)	6.3 (5.8-6.6)	6.7 (5.6-7.1)	0.405
PEEP, cmH_2O	5 (4-6.8)	4.3 (2.3-5.8)	6.5 (5.3-9.0)	0.142
FiO_2	0.4 (0.2-0.5)	0.4 (0.4-0.5)	0.5 (0.4-0.6)	0.139
Peak - PEEP, cmH_2O	17.3 (12.5-19.8)	20.8 (18.5-35)	16.5 (12.6-18)	0.114
Kidney failure	0	1 (8.3)	1 (14.3)	0.375
Inotropes (>12 hrs)	0	1 (8.3)	2 (28.6)	0.089
Vasopressors (>12 hrs)	1 (6.7)	2 (16.6)	3 (42.8)	0.116
Neuromuscular blockade (>12 hrs)	1 (6.7)	1 (8.3)	1 (14.3)	0.839
Systemic corticosteroids (>24 hrs)	1(6.7)	3(25)	2(29.7)	0.322
Failed extubation	3 (100)	0	0	0.124
Duration of MV, hours	134 (88-165)	67.5 (55.2-164.8)	119 (72-148)	0.299
PICU length of stay, days	7 (4-12)	6 (3.3-8.8)	6 (5-9)	0.759
Mortality	0	2 (16.7)	1 (14.3)	0.269
