# Supplementary material for: The impact of critical illness on the expiratory muscles and the diaphragm assessed by ultrasound in mechanical ventilated children
Source: Ann Intensive Care. 2020 Aug 27;10:115. doi: 10.1186/s13613-020-00731-2 (PMC7450159; doi:10.1186/s13613-020-00731-2)
Supplement: Supplementary file 8 — Additional file 8: Thickening fraction at end-expiration of the expiratory abdominal muscles is expressed in percentage (%). Patients are divided in 3 groups according to the change in expiratory muscles thickness during the first 4 days of MV. The number of patients with changes in thickness of >10% decrease, ≤10% change or with >10% increase in thickness for the different muscles’ groups were as follow: 15, 12, 7 (total expiratory muscles); 12, 9, 13 (OE), 16, 12, 6 (OI); 17, 6, 11 (TA); 4, 15, 15 (RA). OE = m. obliquus externa, OI = m. obliquus interna, TA = m. transversus abdominis, RA = m. rectus abdominis, MV = mechanical ventilation. [file 13613_2020_731_MOESM8_ESM.pdf]

Additional file 8

Thickening fraction over first 4 days of the expiratory muscles for the overall study group and per patient subgroup based on changes in thickness in first 4 days

	Overall study population (*n* =34)	>10% decrease	≤10% change	>10% increase	*P* Value
Baseline measurements					
Total expiratory muscles (OE, OI, TA)	3.26 (1.84-5.10)	3.39 (3.13-6.42)	3.20 (0.97-4.43)	3.18 (1.88-3.73)	0.428
m. Obliquus externa	3.67 (0-7.12)	4.02 (0.42-9.12)	0 (0-5.24)	3.73 (0-8.41)	0.325
m. Obliquus interna	4.61 (1.63-7.49)	3.96 (1.69-5.62)	7.36 (2.33-8.87)	4.28 (1.11-7.36)	0.371
m. Transverse abdominis	3.66 (2.28-7.34)	5.8 (2.53-7.73)	3.46 (2.8-6.17)	2.49 (0.87-5.78)	0.225
m. Rectus abdominis	2.37 (0.62-5.33)	2.11 (0.39-4.64)	4.45 (1.97-6.38)	0.92 (0-4.22)	0.221
Last measurements					
Total expiratory muscles (EO, OI, TA)	4.52 (1.66-6.66)	5.88 (1.82-6.71)	4.07 (1.67-6.06)	4.31 (1.17-13.02)	0.844
m. Obliquus externa	2.29 (0-5.68)	2.29 (0-6.74)	5.15 (1.94-7.35)	0 (0-4.22)	0.183
m. Obliquus interna	4.78 (4.41-11.26)	6.95 (2.56-12.49)	3.54 (0.14-8.46)	1.89 (0-11.14)	0.271
m. Transverse abdominis	4.33 (2.45-7.83)	4.54 (2.74-7.03)	4.9 (1.84-9.35)	3.86 (2.08-9.66)	0.971
m. Rectus abdominis	1.33 (0-4.0)	3.76 (1.27-6.57)	1.73 (0-5.57)	0.73 (0-3.83)	0.328
Average over first 4 days					
Total expiratory muscles (EO, OI, TA)	4.91 (2.76-6.66)	4.67 (3.46-6.61)	6.39 (2.09-8.18)	2.76 (1.30-5.87)	0.274
m. Obliquus externa	4.18 (2.87-5.42)	4.83 (3.70-6.49)	3.59 (3.0-4.70)	3.51 (2.26-5.61)	0.262
m. Obliquus interna	5.95 (3.30-8.87)	7.39 (4.06-9.75)	5.86 (1.40-7.67)	4.91 (0.37-7.04)	0.162
m. Transverse abdominis	6.13 (2.99-9.06)	4.86 (3.09-8.91)	5.05 (2.53-7.68)	6.81 (2.11-9.62)	0.769
m. Rectus abdominis	2.41 (1.61-4.10)	2.67 (2.5-4.09)	2.1 (1.67-4.23)	2.09 (1.1-4.06)	0.572
